# Detection of aerobic bacterial pathogens associated with early embryonic death in pregnant New Zealand female Rabbits in Egypt

**DOI:** 10.14202/vetworld.2021.986-995

**Published:** 2021-04-24

**Authors:** Heba Roshdy, Azhar G. Shalaby, Ahmed Abd Elhalem Mohamed, Heba Badr

**Affiliations:** Reference Laboratory for Veterinary Quality Control on Poultry Production, Animal Health Research Institute, Agricultural Research Center, Nadi El-Seid Street, Dokki P.O. Box246, Giza 12618, Egypt

**Keywords:** early embryonic death, *Escherichia coli*, Listeria, Rabbits, *Salmonella*, *Staphylococcus aureus*

## Abstract

**Background and Aim:**

Rabbits are a highly sensitive species and susceptible to various bacterial pathogens that may be causative agents for early embryonic death. This study aimed to explore the administration of different bacterial agents in does suffering from early embryonic death. Furthermore, identification of genes associated with virulence was performed to identify the phenotypic and genotypic antimicrobial resistance patterns that may increase the virulence of pathogens and lead to early embryonic death.

**Materials and Methods:**

We isolated and identified bacterial agents in 106 samples from live and dead female rabbits that had undergone early embryonic death, including liver and intestine tissue, aborted fetuses, discharges, and vaginal swabs. Conventional polymerase chain reaction (PCR) was conducted to confirm the identity of the isolated bacterial strains and their virulence. Moreover, antibiotic resistance was studied phenotypically and genotypically.

**Results:**

We isolated *Escherichia coli*, *Salmonella*, *Staphylococcus aureus*, *Pasteurella multocida*, and *Listeria monocytogenes*. PCR confirmed typical identification except in *P. multocida*, which was confirmed as *Gallibacterium* spp. in some cases. The final percentage of detection was 34%, 30.2%, 16.9%, 13.2%, and 11.3%, respectively. Virulence properties were investigated using different designated genes. All *Salmonella* strains harbored *invA*, *stn*, *avrA*, and *ompf* genes, while the *sopE* gene was identified in 31.25%. *E. coli* strains harboring the *iss* gene lacked the shiga toxin (*stx1*) gene. *L. monocytogenes* and *S. aureus* strains harbored the hemolysin gene (66.7% and 33.4%, respectively). Multidrug resistance was detected phenotypically and genotypically in most strains. Each bacterial pathogen had a different antibiotic resistance profile.

**Conclusion:**

Multiple bacterial species may contribute to early embryonic death in does. Furthermore, the combined infection could be the main cause of early embryonic death. Thus, monitoring programs should bear this in mind and focus on the early detection of these bacterial agents in female rabbits to avoid embryonic death.

## Introduction

Rabbit meat is a source of protein of animal origin and has a high yield [1]. However, rabbits are highly susceptible to various bacterial infections that can be harmful to their health [2]. Early embryonic death refers to the expulsion of a living or dead fetus from the uterus before completing gestation and is usually caused by agents affecting the fetus, its membranes, or both. There are many reasons for fetal death and early embryonic deaths in rabbits, including nutrient deficiency, bacteria, viruses, mycotic and protozoal organisms, toxic agents, and endocrine disorders [3]. *Escherichia coli* is part of the normal microflora colonizing the gastrointestinal tract of rabbits, and certain strains have been associated with intestinal or extra-intestinal infections. Colibacillosis, a common disease in rabbits, is one of the major infectious diseases that endanger the rabbit breeding industry [4]. Virulence factors related to the pathogenicity of extra-intestinal infections of *E. coli* are numerous and have a wide range of activities, from those related to bacteria colonization to those related to virulence, including adhesions, toxins, and iron acquisition factors [5].

*Salmonella* spp. infection in rabbits is characterized by diarrhea, septicemia, and rapid death, and pregnant does commonly abort [6]. *Staphylococcus aureus* is one of the common pathogens causing inflammation in the uterus and abscess or dermatitis in rabbits that are a cause of mortality in young and newly-born rabbits [7]. Does that test positive *Pasteurella multocida*, especially from nasal discharge, often is positive for *P. multocida* from vaginal discharge [6], with commonly observed clinical signs, such as discharge from urogenital opening. Other signs include depression and inappetence with severe emaciation and variable mortality rates [8].

*Listeria monocytogenes* is transmitted by ingestion or inhalation. The infection causes septicemia in young rabbits; meningoencephalitis in adults; and metritis, spontaneous abortion, fetal or neonatal death, and fetal mortality in pregnant does [6]. Antimicrobials drugs are used as preventive measures, as prophylactic treatment during outbreaks of infectious bacterial diseases, and as growth promoters [9]. However, defective policies induce resistance in most pathogens, even in the commensal bacterial of individuals or groups [10].

The significance of this study is its focus on identifying bacterial pathogens that cause early embryonic death in does. This study aimed to explore the administration of different bacterial agents in does suffering from early embryonic death. Furthermore, identification of genes associated with virulence was performed to identify the phenotypic and genotypic antimicrobial resistance patterns that may increase the virulence of pathogens and lead to early embryonic death.

## Materials and Methods

### Ethical approval

Handling of rabbits during sample collection was in accordance with the regulations for the care and welfare of examined animals and approved by the Animal Care Committee of the Animal Health Research Institute.

### Study period and location

The samples collected from January 2018 to August 2019. The sensitivity, serotyping, and PCR were conducted till May 2020 at Reference Laboratory for Veterinary Quality Control on Poultry Production.

### Sample collection

We collected 106 samples from New Zealand female rabbits (does) with aborted fetuses as follows: 20 vaginal swabs from live rabbits who had aborted fetuses, 60 samples from freshly dead does (20 uterine secretions, 24 liver samples, and 16 intestine samples), and 26 dead fetuses aborted by the does Table-1. Samples were collected from animals presented to the Reference Laboratory for Veterinary Quality Control on Poultry Production laboratory under aseptic conditions.

**Table 1:** Type of examined samples.

Sample	Organs	No. of examined samples
Live disease does	Vaginal swabs	20
Freshly dead does	Uterine secretions	20
	Liver	24
	Intestine	16
Aborted fetus	Fetus	26
Total		106

### Bacterial isolation

Samples were examined bacteriologically to identify the different bacterial species that may have caused the cases of abortion, such as *E. coli*, *Salmonella*, *S. aureus*, *Pasteurella* spp., *and L. monocytogenes*.

#### Isolation and identification of E. coli

*E. coli* was isolated and identified according to Lee and Nolan [11]. Briefly, all the collected samples were pre-enriched in buffered peptone water (Oxoid, UK) and incubated aerobically at 37°C for 24 h. Then, a loopful of the broth culture was inoculated onto MacConkey agar (Neogen, US) and eosin methylene blue agar (LabM, UK) plates and reincubated at 37°C for 24 h. The isolated colonies were identified morphologically and biochemically (oxidase strips and triple sugar iron agar from Oxoid; urea, Simmon citrate agar, and peptone water from LabM; and Kovac reagent from HiMedia, India).

#### Serotyping of E. coli isolates

Slide agglutination was used for serotyping the *E. coli* isolates according to their somatic (O) antigens using an *E.coli* antiserum kit (Denka Seiken Co., Ltd, Japan). First, the fresh culture was mixed with normal saline as a control (negative agglutination). Then, the culture was tested with polyvalent O groups 1-8 and if positive, with the monovalent antisera included in the positive polyvalent group.

#### Isolation and identification of Salmonella spp.

*Salmonella* spp. were isolated and identified, according to ISO 6579-1 [12]. Briefly, the samples were pre-enriched in buffered peptone water (Oxoid) and incubated at 37°C for 16-18 h. Then, 0.1 μL of the incubated pre-enrichment medium was transferred to modified semisolid Rappaport–Vassiliadis medium (Oxoid) and incubated at 41.5°C for 24 h. Furthermore, 1 μL of the incubated pre-enrichment medium was transferred to Muller–Kauffmann tetrathionate-novobiocin broth (Oxoid), which was incubated aerobically at 37°C for 24 h. Following incubation, the cultures were onto xylose lysine deoxycholate (Oxoid) and Hektoen enteric agar (Liofilchem, Italy) agar plates and incubated aerobically at 37°C for 24 h. The typical colonies were selected and identified by biochemical tests using urea agar, triple sugar iron agar, and lysine iron agar (Liofilchem).

#### Serotyping of isolated Salmonella species

We performed serotyping of the isolated *Salmonella* species according to ISO 6579-3 [13] using the slide agglutination method with *Salmonella* antiserum (Sifin Co., Germany). First, the fresh culture was mixed with normal saline as a control (negative agglutination). Then, the culture was tested with polyvalent O groups followed by polyvalent flagellar (H) groups, and if positive, with the monovalent O and H antisera to determine the serotype of the *Salmonella* strains according to the Kauffman–White classification [14].

#### Isolation and identification of S. aureus

The isolation and identification of *S. aureus* were performed according to standard methods [15,16]. Inoculated pre-enriched buffer was plated on Baird Parker agar (Oxoid) and incubated at 37°C for 24-48 h. The isolated colonies were identified morphologically (black colony surrounded with double zones indicating tellurite reduction with lipase activity), microscopically (Gram-positive grape-like structure), and biochemically (positive for the slide catalase test, mannitol fermentation, tube coagulase test, and acetoin production [Voges–Proskauer test] and oxidase-negative).

#### Isolation and Identification of Pasteurella spp.

Isolation and identification of *Pasteurella* spp. were performed as per Glisson *et al*. [17]. Briefly, a loopful of each sample was inoculated under aseptic conditions on 7% sheep blood agar plates (LabM) and incubated for 24 h at 37°C. Dew drop-like mucoid non-hemolytic colonies were subjected for further biochemical identification oxidase and indole positive and microscopy showing Gram-negative coccobacilli with characteristic staining bipolarity[17]. MacConkey agar media were used to differentiate *P. multocida* from other members of the Pasteurellaceae family.

#### Isolation and identification of L. monocytogenes

*L. monocytogenes* was isolated and identified, according to ISO 11290-1 [18]. Briefly, the samples were inoculated into a selective primary enrichment medium (half Fraser broth, Oxoid) and incubated at 30°C for 24 h, followed by inoculation of 0.1 μL of the incubated broth into a full-strength secondary liquid enrichment medium (full Fraser broth, Oxoid). After incubation at 37°C for 24 h, the broth was streaked on Agar *Listeria* according to Ottaviani and Agosti (ALOA) medium and Oxford agar (LabM and Oxoid) and incubated at 37°C for 24-48 h. The typical colonies were blue-green surrounded with or without an opaque halo on ALOA agar and olive green with a black halo on Oxford agar. Then, the *Listeria* strains were identified using API strips (BioMérieux, France).

### Antimicrobial sensitivity test

The antibiograms of all isolates were performed by the disk-diffusion method [19] against 11 antibiotics (Oxoid), namely, ampicillin (AMP10), cefotaxime (CTX30), ceftriaxone (CRO30), chloramphenicol (C30), ciprofloxacin (CIP5), doxycycline (DO30), gentamycin (CN10), norfloxacin (NOR10), streptomycin (S10), tetracycline (T30), and trimethoprim-sulfamethoxazole (SXT25), and interpreted, according to the Clinical and Laboratory Standards Institute guidelines [20].

### Molecular assessment

DNA was extracted from culture broth using a QIAamp DNA Mini Kit (Qiagen, Germany, GmbH Catalogue no.51304). The extracted DNA was used in subsequent polymerase chain reaction (PCR) assays for species confirmation and to detect genes responsible for virulence and antimicrobial agent resistance PCR was performed in a final volume of 25 μL that contained 12.5 μL of EmeraldAmp MAX PCR Master Mix (Emerlad, Amp GT (2× premix), Japan), 1 μL of each primer at concentrations of 20 pmol, 4.5 μL of diethylpyrocarbonate water, and 6 μL of the DNA template. The reaction was performed in a Biometra thermal cycler, T3000 (Germany). The oligonucleotide primers (Table-2) [21-38] were supplied by Metabion, Germany.

**Table 2:** Primers sequences, target genes, amplicon sizes, and cycling conditions.

Organism	Target genes	Primers sequences	Amplified segment (bp)	Annealing	Reference
*Escherichia coli*	*phoA*	CGATTCTGGAAATGGCAAAAG CGTGATCAGCGGTGACTATGAC	720	58	[21]
	*Iss*	ATGTTATTTTCTGCCGCTCTG CTATTGTGAGCAATATACCC	266	54	[22]
	*st×*1	ACACTGGATGATCTCAGTGG CTGAATCCCCCTCCATTATG	614	58	[23]
	*tet* (A)	GGTTCACTCGAACGACGTCA CTGTCCGACAAGTTGCATGA	576	50	[24]
	*bla* TEM	ATCAGCAATAAACCAGC CCCCGAAGAACGTTTTC	516	54	[25]
	*qnr*S	CTATTGTGAGCAATATACCC TAAATTGGCACCCTGTAGGC	516	55	[26]
	*aad*1	TATCAGAGGTAGTTGGCGTCAT GTTCCATAGCGTTAAGGTTTCATT	484	54	[24]
*Salmonella*	*inv*A	GTGAAATTATCGCCACGTTCGGGCAA TCATCGCACCGTCAAAGGAACC	284	55	[27]
	*stn*	TTG TGT CGC TAT CAC TGG CAA CC ATT CGT AAC CCG CTC TCG TCC	617	59	[28]
	*avr*A	CCT GTA TTG TTG AGC GTC GTC TGG AGA AGA GCT TCG TTG AAT GTC C	422	58	[29]
	*sop*E	ACT CCT TGCACA ACC AAA TGC GGA TGT CTT CTG CAT TTC GCC ACC	422	58	
	*omp*F	CCTGGCAGCGGTGATCC TGGTGTAACCTACGCCATC	519	50	[30]
	*qac* ED1	TAA GCC CTA CACAAA TTG GGA GAT AT GCC TCC GCA GCG ACT TCCACG	362	58	[31]
*Staphylococcus aureus*	*clf*A	GCAAAATCCAGCACAACAGGAAACGA CTTGATCTCCAGCCATAATTGGTGG	638	55	[32]
	*hlg*	GAAGTCTGGTGAAAACCCTGA TGAATCCTGTCGCTAATGCC	704	60	[33]
Family *Pasteurellacae*	*Kmt1	ATC-CGC-TAT-TTA-CCC-AGT-GG GCT-GTA-AAC-GAA-CTC-GCC-AC	460	55	[34]
	*ssa	TTCACATCTTCATCCTC TTTTCATCCTCTTCGTC	506	45	[35]
	*16S rRNA-23S rRNA	TATTCTTTGTTACCARCGG GGTTTCCCCATTCGG	1032	55	[36]
*Listeria monocytogens*	*16S RNA*	ggA CCg ggg CTA ATA CCg AAT gAT AA TTC ATg TAg gCg AgT TgC AgC CTA	1200	60	[37]
	*hlyA*	GCA-TCT-GCA-TTC-AAT-AAA-GA TGT-CAC-TGC-ATC-TCC-GTG-GT	174	50	[38]

* *Kmt*1 (*P.multocida*),

* *ssa* (*Mannheimia*) and

* *16S rRNA-23S rRNA* (*Gallibacteria*)

The PCR products were separated by electrophoresis according to Sambrook *et al*. [39] on a 1% agarose gel (Applichem, Germany, GmbH) in 1× TBE buffer at room temperature using a gradient of 5 V/cm. Each well was loaded with 15 μL of the PCR product. A Gelpilot 100 bp (Qiagen) ladder was used to determine the fragment sizes. The gel was photographed by a gel documentation system (Biometra BDA digital, Germany), and the data were analyzed using gel documentation system (Alpha Innotech, Biometra Germany) and the computer software (automatic image capture software protein simple formerly Cell Bioscience, USA).

## Results

### Bacteriological results

The 106 samples of female rabbits suffering from early embryonic death were examined for *E. coli*, *Salmonella, S. aureus*, Pasteurellaceae, and *Listeria* isolation, and mixed infections were recognized. *E. coli*, *Salmonella* spp., *S. aureus, Pasteurella* spp., *and Listeria* spp. were identified in 36 (34%), 32 (30.2%), 18 (16.9%), 14 (13.2%), and 12 (11.3%) samples, respectively (Table-3). Different serotypes of *E. coli* were detected (O125, O111, O114, O142, O167, O152, and O119). Different *Salmonella* serotypes were also identified; most serovars were *S*. Enteritidis, followed by *S*. Typhimurium, *S*. Give, *S*. Stuttgart, and then *S*. London, *S*. Derby, *S*. Elisabethville, and *S*. Suberu (Table-4).

**Table 3:** Incidence of pathogenic microorganisms isolated from live and freshly dead pregnant female rabbit and its aborted fetus.

Samples	No. of isolated microorganisms				

	*Escherichia coli*	*Salmonella*	*S. aureus*	*Pasteurellacae*	*Listeria*
Vaginal swabs (diseased does)	8	8	2	0	4
Uterine secretions (dead does)	4	8	8	2	4
Liver	14	6	2	4	0
Intestine	4	2	0	0	0
Aborted fetus	6	8	6	8	4
Total	36	32	18	14	12
% from total -examined (106)	34%	30.20%	16.90%	13.20%	11.30%

**Table 4:** Prevalence of *Escherichia coli* and *Salmonella* serotypes from examined samples.

Samples	Organs	*Escherichia coli* Serotypes(No = 36)	*Salmonella* serovars (No = 32)
Live disease	Vaginal swabs	2 (O125), 2 (O111),	(4) *S.* Enteritidis
		2 (O114),	(2) *S.* Suberu
		2 (O142)	(2) *S.* Elisabeth ville
Freshly dead	Uterine secretions	4 (O125)	(2) *S.* Enteritidis
			(2) *S.* Typhimurium
			(2) *S.* Give
			(2) *S.* Stuttgart
	Liver	10 (O125),	(2) *S.* Enteritidis
		2 (O167), 2 (O152)	(2) *S.* Typhimurium
			(2) *S.* London
	Intestine	4 (O125)	(2) *S.* Stuttgart
Aborted fetus	Fetus	4 (O125), 2 (O119)	(4) *S.* Enteritidis
			(2) *S.* Derby
			(2) *S.* Give

The PCR results confirmed 18 (16.9%) isolates as *S. aureus* using the *clf*A gene. In addition, although 14 *Pasteurella* spp. were identified as *P. multocida* by morphologic, microscopic, and biochemical characteristics, confirmation by PCR assay using three different genes identified 12 of the 14 strains as *Gallibacterium* spp (*Gallibacterium* is a genus of Pasteurellaceae family).

Regarding the confirmation of characterized colonies on ALOA agar by API, four typical isolates were identified as *L. monocytogenes* (identified no. 6510) and confirmed by PCR using *16S RNA* for *L. monocytogenes*, and the remaining eight isolates were identified as *Listeria grayi* (identified no. 3520).

### Antimicrobial results

The results of antimicrobial sensitivity testing all isolates of *E. coli*, *Salmonella*, *S. aureus*, *Pasteurella* spp., and *Listeria* spp. are shown in Table-5. The *E. coli* isolates were highly resistant to tetracycline (94.4%) and doxycycline (88.9%). They were also resistant to trimethoprim-sulfamethoxazole (55.6%); norfloxacin, ciprofloxacin, and streptomycin (66.7%); and ampicillin and chloramphenicol (50%). On the other hand, *Salmonella* only showed a high resistance for streptomycin (68.7%). Isolates of *S .aureus* showed complete resistance to gentamicin (100%), followed by ampicillin (88.9%), but resistance to ceftriaxone and streptomycin was only 44.4%.

**Table 5:** Interpretation of antimicrobial sensitivity of *Escherichia coli, Salmonella*, *Staphylococcus aureus, Pasteurella* spp., and *Listeria* spp. isolates.

Anti- microbial discs/conc.	*Escherichia coli* No=36 (%)			*Salmonella* No=32(%)			*Staphylococcus aureus* No=18 (%)			*Pasteurella* spp. No=14 (%)			*Listeria* spp. No=12(%)		
				
	R	I	S	R	I	S	R	I	S	R	I	S	R	I	S
AMP^10^	18 (50)	2 (5.6)	16 (44.4)	8 (25)	2 (6.2)	22 (68.8)	16 (88.9)	0	2 (11.1)	14 (100)	0	0	12 (100)	0	0
CTX^30^	10 (27.8)	2 (5.6)	24 (66.7)	0	12 (37.5)	20 (62.5)	2 (11.1)	12 (66.6)	4 (22.3)	6 (42.8)	8 (57.1)	0	0	4 (33.3)	8 (66.7)
CRO^30^	10 (27.8)	2 (5.6)	24 (66.7)	0	8 (25)	24 (75)	8 (44.4)	8 (44.4)	2 (11.1)	10 (71.4)	2 (14.3)	2 (14.3)	0	10 (83.3)	2 (16.7)
C^30^	18 (50)	2 (5.6)	16 (44.4)	4 (12.6)	2 (6.2)	26 (81.2)	0	0	18 (100)	10 (71.4)	4 (28.6)	0	4 (33.3)	2 (16.7)	6 (50)
CIP^5^	24 (66.7)	4 (11.1)	8 (22.2)	2 (6.2)	2 (6.2)	28 (87.6)	0	2 (11.1)	16 (88.9)	4 (28.6)	6 (42.8)	4 (28.6)	6 (50)	2 (16.7)	4 (33.3)
DO^30^	32 (88.9)	2 (5.6)	2 (5.6)	6 (18.8)	0	26 (81.2)	0	0	18 (100)	10 (71.4)	4 (28.6)	0	12 (100)	0	0
CN^10^	6 (16.7)	0	30 (83.1)	4 (12.6)	2 (6.2)	26 (81.2)	18 (100)	0	0	8 (57.1)	2 (14.3)	4 (28.6)	0	0	12 (100)
NOR^10^	24 (66.7)	4 (11.1)	8 (22.2)	2 (6.2)	2 (6.2)	28 (87.6)	0	0	18 (100)	2 (14.3)	6 (42.8)	6 (42.8)	4 (33.3)	2 (16.7)	6 (50)
S^10^	24 (66.7)	2 (5.6)	10 (27.8)	22 (68.7)	10 (31.3)	0	8 (44.4)	10 (55.6)	0	12 (85.7)	2 (14.3)	0	4 (33.3)	0	8 (66.7)
T^30^	34 (94.4)	0	2 (5.6)	6 (18.7)	0	26 (81.2)	0	2 (11.1)	16 (88.9)	12 (85.7)	0	2 (14.3)	8 (66.7)	0	4 (33.3)
SXT^25^	20 (55.6)	0	16 (44.4)	0	0	32 (100)	0	0	18 (100)	10 (71.4)	0	4 (28.6)	4 (33.3)	2 (16.7)	6 (50)

R=Resistance, I=Intermediate, S=Sensitive, Amp=Ampicillin, CTX=Cefotaxime, CRO=Ceftriaxone, C=Chloramphenicol, CIP=Ciprofloxacin, DO=Doxycycline, CN=Gentamycin, NOR=Norfloxacin, S=S treptomycin, T=Tetracycline and SXT=Trimethoprim-sulfamethoxazole

*Pasteurella* spp. showed a wide range of resistance to ampicillin (100%); tetracycline and streptomycin (85.7%); and ceftriaxone, chloramphenicol, doxycycline, and trimethoprim-sulfamethoxazole (71.4%); but only 57.1% to gentamycin. Finally, *Listeria* spp. exhibited complete resistance to both ampicillin and doxycycline (100%), followed by tetracycline (66.7%) and ciprofloxacin (50%), while the percentage of resistance was 33.3% to norfloxacin, chloramphenicol, and streptomycin.

### PCR results

All the isolated strains underwent confirmation by screening for common genes using conventional PCR: *phoA* for *E. coli*, *invA* for *Salmonella*, *clfA* for *S. aureus*, and a variety of genes for the Pasteurellaceae family (*kmt1* for *P. multocida*, *ssn* for *Mannheimia*, and 16S and 23S RNA for *Gallibacterium*), and 16S RNA for *L. monocytogenes*.

We used conventional PCR techniques with specific primers to detect virulence genes in *E. coli*, *Salmonella*, *S. aureus*, and *L. monocytogenes*. The genes were selected if they occurred at a high frequency in the bacteria. The results showed that the *E. coli* strains incorporated the serum resistant gene (*iss*) but lacked the shiga toxin (*stx*) gene. All *Salmonella* strains carried the enterotoxin gene *stn*, invasion genes *invA* and *avrA*, and the outer membrane protein gene *ompF*, but the *sopE* gene was only present in 31.25% of isolates. However, the hemolysin gene (*hly*) was found in 66.7% of *S. aureus* strains and the hemolysin gene (*hlg*) in 33.3% of *Listeria* spp. strains (Table-6).

**Table 6:** Polymerase chain reaction results for the examined virulence genes for different bacterial culture.

Organism	Examined Genes	Positive/Total (%)
*Escherichia coli*	*Iss*	36/36 (100%)
	*St × 1*	0/36 (0%)
*Salmonella*	*stn*	32/32 (100%)
	*invA*	32/32 (100%)
	*avrA*	32/32 (100%)
	*ompf*	32/32 (100%)
	*sopE*	10/32 (31.25%)
*S. aureus*	*hly*	12/18 (66.7%)
*Listeria* spp.	*hlg*	4/12 (33.3%)

**Figure-1:**
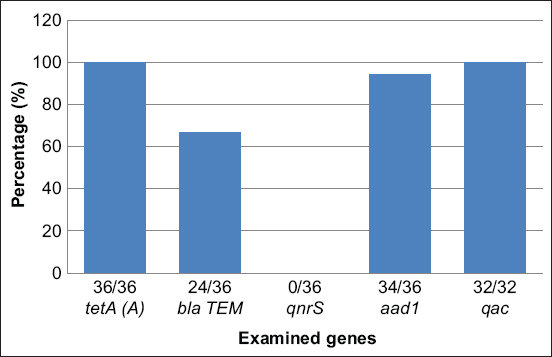
Results for antimicrobial resistant genes of *Escherichia coli* and *Salmonella* by polymerase chain reaction technique.

The genotypic detection for antimicrobial resistance genes was performed for *E. coli* and *Salmonella* strains because these isolates were present in a high percentage of the isolates (34% and 30.2%, respectively) and showed different phenotypic patterns of antimicrobial resistance according to the presence of the selected screening genes Figure-1. The *E. coli* strains harbored genes against many groups of antimicrobial agents such as tetracycline (*tetA* (*A*) gene, 100%), streptomycin (*aad1* gene, 94.4%, 34/36), and b lactamase (*bla TEM* gene, 66.7%, 24/36, but lacked the quinolone resistance gene (*qnrS*) although a percentage resistance to quinolone was phenotypically observed. All *Salmonella* strains had the quinolone-resistant gene *(qac)* but a low percentage of quinolone resistance phenotypically.

## Discussion

In general, rabbit farms are considered the fastest growing industry for their income, which is derived from meat, fur, or the use of rabbits as experimental animals for laboratory trials. Thus, it is crucial to determine important rabbit diseases to avoid economic loss and zoonotic threats [40]. For this reason, we focused on cases of aborted female does because reproductive females are the most important link in the production chain and recorded the bacterial pathogens present in different organs. We collected 106 samples from aborted does, which identified *E .coli*, *Salmonella* spp., *S. aureus*, *Pasteurella* spp., and *Listeria* spp. in percentage of 36 (34%), 32 (30.2%), 18 (16.9%), 14 (13.2%), and 12 (11.3%) samples, respectively. Pisoni *et al*. [41] categorized the most frequent lesions in rabbits as affecting the gastroenteric organs, followed by the genitourinary, respiratory, and cutaneous organs. In addition, the presence of *E. coli* was 29.8%, *S. aureus* was 9.3%, and *P. multocida* was 7.9% in rabbit breeding facilities [41].

Colibacillosis is a common disease in rabbits, is a major infectious disease threat to the rabbit breeding industry [4]. *E. coli* was identified as the most prevalent species among bacterial organisms that causing enteritis and mortality in rabbits [42]. Enteropathogenic *E. coli* is the only known class of *E. coli* in rabbits that induces acute intestinal pathology marked by inflammatory lesions of the gut [43]. Our study isolated *E. coli* in 36 of 106 (34%) samples as a single or combined infection. The *E. coli* serotypes were confirmed as O125, O111, O114, O142, O167, O152, and O119, which, with the exception of O119, did not correlate with those of Hamed *et al*. [42], who confirmed *E. coli* serotypes O126, O128, O125, and O119 from diseased rabbits. Another study reported *E. coli* serotypes O55, O128, O126, O119, O78, O44, O111, O114, O26, O75, O103, O145, and O158 as frequently isolated from infected rabbits [44]. In Egypt, the higher isolation percentage of *E. coli* from rabbits was reported by Eid *et al*. [43] (525 of 625, 84%) that belonged to serotypes O153, O125, O27, and O158. Collectively, these findings indicate that a wide range of *E. coli* serotypes in rabbits may cause disease conditions.

Virulent pathogenic bacteria have more tools and abilities to increase their systemic action, escaping from the host’s immune system and withstanding the bactericidal effect of the antimicrobial drugs. Combinations of the virulence and antibiotic resistance genes result in new and uncontrollable bacterial strains with higher morbidity and mortality rates than the original bacteria. Thus, we attempted to identify genes suspected of increasing bacterial virulence. First, we did not detect there was no shiga toxin (*stx*) in any of the *E. coli* isolates, but the *iss* was gene detected in all *E. coli* isolates. This result was similar to that reported by Eid *et al*. [43], who examined different isolates of *E. coli*, none of which were positive for *stx1* or *stx2*. Other authors [45,46] discussed the presence of shiga toxin, which is encoded by genes, and mentioned its ability to attach the intestinal epithelial cells and causing diarrhea.

Salmonellosis is an unusual cause of acute enteritis in rabbits, which is characterized by rapid death and infertility, while pregnant does commonly abort [42]. The samples from does with aborted fetuses in this study showed different *Salmonella* serovars in 32 of 106) (30.2%) samples, including *S*. Enteritidis, *S*. Typhimurium, *S*. Give, *S*. London, *S*. Derby, *S*. Elisabethville, *S*. Suberu, and *S*. Stuttgart. Pisoni *et al*. [42] also recorded a variety of *Salmonella* serovars from weaning rabbits, including *S*. Enteritidis, *S*. Mbadaka, *S*. Heidelburg, *S*. Typhimurium and *S*. Pullorum.

*S*. Enteritidis was the most isolated serovar in our study (12/32), but *S*. Typhimurium was also isolated (4/32). This finding contradicts those of Iman and Lamia [47], who mentioned that the most detected strain from vaginal swabs from aborted cases was *S*. Typhimurium (10/135, 7.40%). Therefore, no clear relationship between specific *Salmonella* serovars was identified as associated with induced abortion.

The virulence properties of *Salmonella* strains were assessed by the presence of different virulence genes, such as *inv*A, *stn*, *avr*A, and *omp*E, which were present in all examined *Salmonella* strains. However, the *sopE* gene was only detected in 31.25% of the *Salmonella* strains. The function of *inv*A (*Salmonella* invasion protein gene) is the attachment of the pathogen to the host in addition to *phoA*, *SopE*, and *avrA*) [27]. The enterotoxin (*stn*) gene was demonstrated as a suitable PCR target for the detection of *Salmonella* strains with most potent biological poisons [48]. Undoubtedly, all isolated *Salmonella* strains in our study carried virulent genes to help in attaching and invading all of the rabbits’ body systems.

*S. aureus* was isolated from intrauterine metritis rabbits of a dead pregnant female [49]. *S. aureus* is a dangerous pathogen, secreting a number of toxins and hemolysins that cause tissue damage [50]. In a sample of 106 rabbits from nine rabbit farms, 18 (16.9%) *S. aureus* strains were detected, of which 12 (66.7%) harbored the hemolysin gene [51]. After studying the mortality rates of these nine rabbit farms, it was suggested that *S. aureus* was an important pathogen causing the death of rabbits on these rabbit farms. In general, *S. aureus* has a combination of virulence factors, which were thought to contribute to the pathogenicity. The presence of virulence genes in *S. aureus* isolates was screened by PCR assays using primers previously reported, including those for hemolysin (hla and hlb). This study highlighted the importance of *S. aureus* in infection or coinfection results in cases of early embryonic death, especially with the high prevalence of this virulence gene.

*Pasteurella* is a normal inhabitant of the upper respiratory tract in a variety of animal species, including rabbits. However, many factors can convert it into a pathogenic form leading to death [52]. In the present study, there were 14 of 106 (13.2%) *Pasteurella* spp. identified as *P. multocida* by morphologic, microscopical, and biochemical characteristics from samples of aborted fetus, followed by liver and then uterine secretions. Identification was confirmed by PCR using three different genes, which identified 12 of the 14 strains as *Gallibacterium* spp.

*P. multocida* was isolated and confirmed from liver samples by Asran *et al*. [53]. PCR represents a suitable tool for genetic characterization of rabbit Pasteurellaceae isolates while the biochemical analysis showed high heterogeneity and in some cases provided unclear results [54]. *Gallibacterium* is a commensal of the upper respiratory tract and the lower genital tract of chickens [55] and in non-avian hosts, including cattle, horse, pigs, sheep, and rabbits [56]. As observed in this study, *Gallibacterium* spp. is also present in aborted rabbits, although its role in the rabbit industry required further investigation.

Listeriosis in rabbits is considered as one of the major disease problems facing rabbit health and breeding in the form of infertility, abortion, or myometrial contraction and high mortality resulting in severe economic losses in rabbit farms [57]. In our study, 12 (11.3%) *Listeria* spp. were identified, but only four strains were *L. monocytogenes*, which were present in samples of aborted fetuses and vaginal swabs. A higher incidence of *L. monocytogenes* was reported by Hatab and El-Latif [58], with a percentage of 30% and 20% from aborted dead and aborted live does, respectively. However, the incidence of *L. monocytogenes* was reported as 21.73% and 31.66% from aborted does and dead fetuses, respectively, as mentioned by Moursi *et al*. [57] during an examination of rabbit farms in Ismailia of rabbits suffering from encephalitis and abortion.

Because most of the isolated strains in our study were *Salmonella* and *E. coli*, it prompted us to seek suitable treatment methods and acquired resistance genes, especially after detection of the virulence pattern of bacterial species such as *Salmonella*, *E. coli*, *S. aureus*, and *L. monocytogenes*.

Antimicrobial resistance is a concern in human and veterinary medicines as being implicated in the failure of treatment protocols. Therefore, the proper determination of the resistance patterns represents a prerequisite for adapting successful control measures [59]. In this study, all the isolated strains related to different bacterial species were examined using the disk-diffusion method with different antimicrobials. A wide range of antimicrobial resistance was noted. A high resistance pattern in *E. coli* isolates was noted against tetracycline, doxycycline, and trimethoprim-sulfamethoxazole (94.4%, 88.9%, and 55.6%, respectively), and the representative gene for *tetA* was present in all strains of *E. coli*. However, the prevalence of resistance against norfloxacin and ciprofloxacin was 66.7%, but the genes regulating quinolone resistance (*qnrs*) were absent in all *E. coli* strains. Furthermore, the *aad1* gene was detected in 94.4% of isolates, but only 66.7% showed phenotypic resistance to streptomycin. The resistance gene *bla* Tem was present in 66.7%, and the lowest phenotypic resistance percentage was 50% for ampicillin and chloramphenicol. Furthermore, Zhao *et al*. [4] reported resistance rates of 55 *E. coli* strains against tetracycline and ampicillin as 78.2% and 65.5%, respectively. Landoni and Albarellos [60] mentioned that the wide range of antimicrobial agents, such as b-lactams (penicillins and cephalosporin), aminoglycosides, tetracycline, sulfonamides, and fluoroquinolones was used in Colibacillosis treatment. The mechanism of tetracycline resistance was mediated through the efflux pump and encoded for a group of genes (*tetA*, *tetB*, *tetC*, *tetD*, *tetE*, and *tetG*) [61]. The difference in phenotypic and genotypic results revealed that genotypic detection is not the best indicator of resistance.

*Salmonella* revealed high resistance only for streptomycin (68.7%) and low resistance to tetracycline and quinolones, although the quinolone-resistant gene (*qac*) was present in all examined *Salmonella* strains. Gram-negative bacteria were reported as most frequently found in combination with genes coding for resistance to aminoglycosides, chloramphenicol, sulfonamides, trimethoprim, and b-lactams [62]. In contrast, Iman and Lamia [47] reported that rabbit is a potential reservoir for salmonellosis and thecommon *Salmonella* serotype was *S*. Typhimurium DT10 with multi-resistant phenomena emergent in the circulating *Salmonella* strains from rabbit sources. In addition, animal breeding could represent a possible reservoir of antibiotic resistance and virulent bacteria, as observed in rabbits [63].

*S. aureus* is pathogenic and can develop antibiotic resistance, resulting in high morbidity and mortality [51]. In the present study, the isolated *S. aureus* strains showed high resistance for gentamycin (100%) and ampicillin (88.9%) but only 44.4% resistance for ceftriaxone and streptomycin and were highly sensitive to ciprofloxacin and trimethoprim and sulfamethoxazole. Hamed and Youssef [64] found that all *S. aureus* strains isolated from rabbits in Egypt were multidrug-resistant to trimethoprim and sulfamethoxazole, and gentamicin and ampicillin, and all strains were sensitive to ciprofloxacin. This finding showed the different pattern of the bacteria toward antimicrobial resistance.

Our study revealed a wide range of the resistance of *Pasteurella* spp. as ampicillin (100%); tetracycline and streptomycin (85.7%); ceftriaxone, chloramphenicol, doxycycline and trimethoprim-sulfamethoxazole (71.4%); and gentamycin (57.1%). In contrast, Wang *et al*. [65] revealed streptomycin, gentamycin, and ceftriaxone with resistance rates of 27.80%, 15.61%, and 2.44%, respectively. Furthermore, Mohamed *et al*. [66] stated that *Pasteurella* spp. only showed resistance against streptomycin and a wide range of antibiotic susceptibility for norfloxacin, ciprofloxacin, doxycycline, and gentamycin.

*Listeria* spp. showed resistance to ampicillin and doxycycline (100%), tetracycline (66.7%), and ciprofloxacin (50%), but only 33.3% resistance to norfloxacin, chloramphenicol, and streptomycin. Hamed *et al*. [42] reported that the treatment with gentamicin and ofloxacin showed progressive cure for experimentally infected rabbits with an *L. monocytogenes* strain sensitive to ofloxacin, erythromycin, gentamicin, norfloxacin, and ciprofloxacin. Furthermore, Moursi *et al*. [57] reported that *L. monocytogenes* was highly sensitive to ampicillin, tetracycline, doxycycline, trimethoprim, and gentamycin and moderately sensitive to ciprofloxacin and chloramphenicol but resistant to streptomycin and cefotaxime.

## Conclusion

Our study illustrates that *Salmonella* is a common pathogen as a single cause of early embryonic death, followed by *E. coli*, *S. aureus*, *Pasteurella*, and *Listeria*, respectively. On the other hand, other cases showed mixed pathogens that were considered the cause of abortion and consisted of combined infection with two, three, or four bacteria, mostly with *Salmonella* or and *E. coli*, while 24 samples were negative for bacterial isolation. Further investigation is necessary to identify the main cause of early embryonic death and to confirm our results, which suggest that the different etiological agent(s) were incorporated and may play a role in the initiation of abortion in does.

## Authors’ Contributions

HB, HR, and AGS designed the study. HB, HR, AGS, and AAEM performed the research and drafted the manuscript. HB and AGS analyzed the data. HB and AGS revised and finalized the manuscript for submission. All authors read and approved the final manuscript.
